# The Underlying Rab Network of MRGPRX2-Stimulated Secretion Unveils the Impact of Receptor Trafficking on Secretory Granule Biogenesis and Secretion

**DOI:** 10.3390/cells13010093

**Published:** 2024-01-01

**Authors:** Pia Lazki-Hagenbach, Elisabeth Kleeblatt, Mitsunori Fukuda, Hydar Ali, Ronit Sagi-Eisenberg

**Affiliations:** 1 Department of Cell and Developmental Biology, Faculty of Medicine, Tel Aviv University, Tel Aviv 69978, Israel; piahagenbach@gmail.com (P.L.-H.); ele.kleeblatt@web.de (E.K.); 2 Laboratory of Membrane Trafficking Mechanisms, Department of Integrative Life Sciences, Graduate School of Life Sciences, Tohoku University, Aobayama, Aoba-ku, Sendai 980-8578, Miyagi, Japan; nori@tohoku.ac.jp; 3 Department of Basic and Translational Sciences, School of Dental Medicine, University of Pennsylvania, Philadelphia, PA 19104, USA; alih@upenn.edu; 4 Sagol School of Neuroscience, Tel Aviv University, Tel Aviv 6997801, Israel

**Keywords:** mast cells, MAS-related G-protein-coupled receptors, MRGPRX2, RBL-2H3, Rab GTPase, degranulation

## Abstract

MRGPRX2, the human member of the MAS-related G-protein-coupled receptors (GPCRs), mediates the immunoglobulin E (IgE)-independent responses of a subset of mast cells (MCs) that are associated with itch, pain, neurogenic inflammation, and pseudoallergy to drugs. The mechanisms underlying the responses of MRGPRX2 to its multiple and diverse ligands are still not completely understood. Given the close association between GPCR location and function, and the key role played by Rab GTPases in controlling discrete steps along vesicular trafficking, we aimed to reveal the vesicular pathways that directly impact MRGPRX2-mediated exocytosis by identifying the Rabs that influence this process. For this purpose, we screened 43 Rabs for their functional and phenotypic impacts on MC degranulation in response to the synthetic MRGPRX2 ligand compound 48/80 (c48/80), which is often used as the gold standard of MRGPRX2 ligands, or to substance P (SP), an important trigger of neuroinflammatory MC responses. Results of this study highlight the important roles played by macropinocytosis and autophagy in controlling MRGPRX2-mediated exocytosis, demonstrating a close feedback control between the internalization and post-endocytic trafficking of MRGPRX2 and its triggered exocytosis.

## 1. Introduction

Mast cells (MCs) are secretory cells of the immune system, whose strategic location in tissues that interface the external environment, jointly with their responsiveness to multiple stimuli, have marked them as sentinel cells and first responders to external stressors [1]. MCs exert their immune responses, following their activation by external triggers, by the release of a wide spectrum of inflammatory mediators. Part of these mediators, such as histamine and proteases are pre-formed and stored in cytoplasmic secretory granules (SGs) until their regulated release by SG exocytosis [2,3], a process referred to as degranulation. Others, including lipid mediators and cytokines/chemokines, are newly synthesized in response to the cell trigger and released [4]. Jointly, the inflammatory mediators that are released by the activated MCs give rise to both immediate and late phase allergic reactions. The most extensively studied are the immunoglobulin E (IgE)-mediated allergic reactions, which follow MC activation by allergen-mediated crosslinking of allergen-directed IgE antibodies that are bound to the FcεRI, the high-affinity receptor for IgE [5]. However, MCs additionally respond to various innate triggers that activate the cells independently of IgE [6,7]. Among the innate triggers, multiple polycationic compounds activate MCs by binding to MAS-regulated G-protein-coupled receptors (Mrgprs), a family of G-protein-coupled receptors (GPCRs) that are expressed in a subset of MCs. Specifically, in rodents, Mrgprs are expressed in connective tissue MCs (i.e., skin and peritoneal MCs), but not mucosal MCs [8,9], whereas in humans, the receptors are highly expressed in skin, fat, and synovial MCs [10]. The list of Mrgpr agonists includes neuropeptides, such as substance P (SP), which, by activating the MCs, cause neuroinflammation [11], antimicrobial peptides that mediate the host defense functions of MCs [12,13,14], and multiple FDA-approved drugs which, by activating the MCs, cause pseudoallergic reactions that are life threatening and unpredictable [8,15,16]. Mrgprs have therefore gained increased interest.

Unlike the IgE-mediated degranulation, which involves the release of fused SGs by a process known as compound exocytosis [17,18], innate stimulation of Mrgprs results in the rapid secretion of individual SGs [18]. Also, the dependence of degranulation on mitochondria dynamics varies between these types of triggers, whereby inhibition of mitochondria fission enhances IgE-mediated MC activation while it suppresses SP-stimulated MC activation [19]. Finally, we have shown that silencing of GPCR kinase 2 (GRK2) inhibits IgE-mediated degranulation, while it potentiates the release induced by MRGPRX2 ligands [20]. These results indicate that distinct mechanisms underlie IgE- versus Mrgpr-triggered degranulation. However, the precise mechanisms employed by Mrgpr triggers are still not completely understood. In particular, the species-dependent variations that Mrgprs have acquired during evolution [21] have raised the need for specific delineation of the mechanism of action of MRGPRX2, the human member of the Mrgpr family. 

The signaling and function of GPCRs are closely associated with their cellular location [22,23,24]. Moreover, we have shown that SP induces the internalization of MRGPRX2 via both endocytosis and macropinocytosis and stimulates the delivery of MRGPRX2 to the SGs, thus suggesting a possible link between post-endocytic trafficking of MRGPRX2 and SG degranulation [25]. To gain insights into the steps in MRGPRX2 trafficking that may influence its function in stimulating exocytosis, we have screened Rab GTPases, which are endogenously expressed in MCs, for their influence on MRGPRX2-mediated secretion. The latter comprise a family of small GTPases that function as molecular switches, by cycling between GDP-bound, inactive, and GTP-bound, active, conformations [26]. In their active, GTP-bound form, Rabs bind to target membranes, and by their binding of specific effector proteins, including SNAREs, motor proteins, tethering factors, and cargo, they control discrete steps along vesicular trafficking [27]. Here, we show that MRGPRX2 responses are regulated by a unique Rab network that underlines the important role played by macropinocytosis and the autophagic machinery in controlling its responses. 

## 2. Materials and Methods

### 2.1. Antibodies and Reagents

Anti-human MRGPRX2 antibody (cat no. 359002) and anti-HA.11 Epitope Tag antibody (cat no. 901513) were obtained from Biolegend (San Diego, CA, USA). Secondary Alexa Fluor^®^ 647 Goat Anti-Mouse IgG H&L (cat no. ab150115) was obtained from Abcam (Cambridge, UK). Substance P (SP) (cat no. S6883), compound 48/80 (c48/80) (cat no. C2313), DAPI (cat no. D9542) were purchased from Sigma-Aldrich (St. Louis, MO, USA). EIPA (cat no. 3378) were obtained from Tocris Bioscience (Minneapolis, MN, USA). Tetramethylrhodamine (TRITC)-labeled 70 kDa Dextran was purchased from Thermo Fischer Scientific (cat no. D1818, Waltham, MA, USA). 

### 2.2. Plasmids Used in This Study

The following expression plasmids were used in this study: neuropeptide Y (NPY)-mRFP was a kind gift from Dr. U. Ashery (Tel Aviv University, Tel Aviv, Israel), and pEGFP-wild type (WT) Rab constructs have been described previously [28]. Constitutively active (CA) Rab mutants were prepared as previously described [29] and subcloned into the pEGFP-C1. pEGFP-LC3-G120A was a kind gift from Aviva Tolkovsky (University of Cambridge, Cambridge, UK), originally a gift from Tamotsu Yoshimori (Osaka University, Osaka, Japan).

### 2.3. Cell Culture

RBL cells stably expressing N-terminally tagged hemagglutinin (HA) human MRGPRX2 (RBL-MRGPRX2) were previously described [12]. Cells were maintained as adherent cell cultures in low glucose DMEM (cat no. 01-050-1A, Biological Industries, Beit-Haemek, Israel) supplemented with 10% FBS (cat no. 12657, GIBCO, Grand Island, NY, USA), 2 mM L-Glutamine (cat no. 03-020-1A, Biological Industries), 100 μg/mL streptomycin, and 100 U/mL penicillin, 12.5 U/mL nystatin (cat no. 03-032-1B, Biological Industries), and 1 mg/mL of G418 (cat no. A1720, Sigma Aldrich, St. Louis, MO, USA), at 37 °C in a humidified incubator with 5% CO_2_. LAD-2 cells (a kind gift from Dr. A.S. Kirshenbaum and Dr. D. Metcalfe, Laboratory of Allergic Diseases, National Institute of Allergy and Infectious Diseases, National Institutes of Health, Bethesda, MD, USA) were cultured in StemPro-34 (cat no. 10640-019, GIBCO) supplemented with 1× StemPro-34 Nutrient, 2 mM L-Glutamine (cat no. 03-020-1A, Biological Industries), 100 U/mL penicillin and 100 μg/mL streptomycin (cat no. 03-032-1B, Biological Industries), and 100 ng/mL hSCF (cat no. 300-07, Peprotech, Rocky Hill, NJ, USA).

### 2.4. Cell Transfection

Transient transfection of RBL-MRGPRX2 cells was performed as previously described [25]. Briefly, RBL-MRGPRX2 cells (1.5 × 10^7^) were transfected with 30–50 μg cDNA by electroporation at 300 V for 20 ms, using an ECM 830 electroporator (BTX, Holliston, MA, USA). The cells were immediately replated in either 24-well (1 × 10^5^ cells/well) tissue culture dishes for confocal imaging, 24-well (4 × 10^5^ cells/well) tissue culture dishes for secretion assays, or 6-well (0.2 × 10^6^ cells/well) tissue culture dishes for flow cytometry experiments containing growth medium and used within 20–24 h (h) after transfection.

### 2.5. NPY Secretion Assay

Secretion of NPY-mRFP was measured as previously described [25,30]. Briefly, after three washes in Tyrode’s buffer (10 mM HEPES [pH 7.4], 130 mM NaCl, 5 mM KCl, 1.4 mM CaCl_2_, 1 mM MgCl_2_, 5.6 mM glucose, and 0.1% BSA), the cells were either left untreated or triggered with 1 μg/mL c48/80 or 10 μM SP for 30 min in Tyrode’s buffer at 37 °C. In experiments that involved the macropinocytosis inhibitor EIPA, the cells were preincubated with 50 μM EIPA for 30 min, after which the cells were incubated with the triggers in the presence of EIPA for another 30 min. Fluorescence of cell supernatants and cell lysates (200 μL) was measured by using a fluorescence microplate reader (BioTek Synergy H1 Multimode Reader, Agilent, Santa Clara, CA, USA) with excitation at 579 nm and emission at 616 nm. Release of NPY-mRFP is presented as percentage of control. Release is defined as percent secretion untreated subtracted from percent secretion with trigger.

### 2.6. Dextran Uptake Assay

RBL-MRGPRX2 cells (1 × 10^5^ cells/well) were seeded on 13-mm round glass coverslips (cat no. 41001113, 13 mm Ø, thickness 1 round, Glaswarenfabrik Karl Hecht GmbH & Co. KG, Sonderheim, Germany) in 24-well plates, and incubated with 0.1 mg/mL of TRITC-labeled 70 kDa Dextran (cat no. D1818, Thermo Fischer Scientific) in Tyrode’s buffer for 15 min at 37 °C and 5% CO_2_ in the absence or presence of 1 μg/mL c48/80 or 10 μM SP. Cells were washed three times with ice-cold PBS and fixed for 20 min at room temperature (RT) with 4% paraformaldehyde (cat no. 0006750523F1, Bio-Lab Ltd., Jerusalem, Israel). Subsequently, cells were immunostained as detailed under Immunostaining and Laser Confocal Microscopy Analysis. 

### 2.7. Flow Cytometry Analysis

RBL-MRGPRX2 cells were washed three times in Tyrode‘s buffer to remove dead, non-adherent cells and subsequently stimulated at 37 °C and 5% CO_2_ in Tyrode‘s buffer for the desired time periods with vehicle, 1 μg/mL c48/80 or 10 μM SP. In experiments that involved EIPA, the cells were preincubated with 50 μM EIPA for 30 min after which the cells were incubated with the triggers in the presence of EIPA for another 30 min. Cells were then washed three times with ice-cold PBS containing 0.5% BSA (FACS buffer) and detached from the plate by scraping. For analyses of cell surface MRGPRX2, cells were stained for 30 min at 4 °C with anti-human MRGPRX2 antibodies (1:300 dilution). Cells were subsequently washed three times and stained with Alexa Fluor^®^ 647-conjugated Goat Anti-Mouse IgG H&L secondary antibody (1:2000 dilution) for 30 min at 4 °C, in the dark. Cells were washed three times with FACS buffer, subsequently incubated with 1 μM DAPI for 15 min at 4 °C to assess cell viability, and analyzed by flow cytometry using a CytoFLEX LX flow cytometer (Beckman Coulter, Indianapolis, IN, USA). A minimum of 10,000 cells was acquired per sample. Cells were gated on single cells and were additionally gated on GFP-positive cells to gate for transfected cells. Data were analyzed using the FlowJo™ Software Version 10 (Treestar, Ashland, OR, USA). To determine the type of internalization of MRGPRX2, the percentage of internalization via macropinocytosis (MP) was calculated as the difference of internalization in the absence or presence of EIPA. The percentage of endocytosis was calculated by subtracting the percentage of MP from the percentage of total internalization. Negative values are displayed as zero.

### 2.8. Immunostaining and Laser Confocal Microscopy Analysis

RBL-MRGPRX2 (1 × 10^5^ cells/well) were seeded on 13-mm round glass coverslips (cat no. 41001113, 13 mm Ø, thickness 1 round, Glaswarenfabrik Karl Hecht GmbH & Co.KG, Sonderheim, Germany) in 24-well plates, were washed three times in Tyrode’s buffer; the cells were stimulated as indicated in the same buffer at 37 °C and 5% CO_2_. The cells were subsequently washed three times with ice-cold PBS and fixed for 20 min at RT with 4% paraformaldehyde (cat no. 0006750523F1, Bio-Lab Ltd., Jerusalem, Israel) in PBS followed by permeabilization for 15 min with 0.1% Triton X-100, 5% FBS, and 2% BSA diluted in PBS. Subsequently, cells were labeled with mouse anti-HA (1:250 dilution) antibody for 1 h at RT, followed by three washes and a 1-h incubation with Alexa Fluor^®^ 647 Goat Anti-Mouse IgG H&L secondary antibody (1:500 dilution). After washing, cells were mounted in mounting medium (cat no. E18-18; Golden Bridge Life Science, Mukilteo City, WA, USA) and analyzed with a Zeiss LSM 710 confocal microscope equipped with a 63 × 1.4 oil Plan-Apochromat objective. Colocalization of NPY-mRFP with pEGFP-CA RabX was quantified by the Manders’ correlation coefficient with Otsu thresholding [31] using the JaCoP plugin of the extended ImageJ version Fiji (Version 2.9.0/1.53t) [32,33,34]. To determine the SG size and number/cell, images were converted into threshold images and then analyzed using the ImageJ “analyze particles” macro. To determine the Dextran puncta/cell, each cell was outlined using the segmented line tool on the brightfield image and saved as a region of interest (ROI). Next, the ROI was used to analyze the amount of puncta/cell using the ImageJ “analyze particles” macro on the appropriate channel [33]. The number and size of macropinosomes were calculated by drawing the borderline of each macropinosome with the segmented line tool, saved as ROIs. The length of the ROIs was then measured using the extended ImageJ version Fiji [33]. The percentage of SGs in cell tips and periphery as well as the perinuclear clustering of SGs were determined by counting the number of cells out of total cells that exhibited SGs in the tips/rim or the periphery, or at the perinuclear region, respectively.

### 2.9. Statistical Analysis

Data were analyzed using GraphPad Prism Version 8.3.0 for Windows (GraphPad Software, La Jolla, CA, USA). One-way analysis of variance (ANOVA) with repeated measures followed by Bonferroni or Dunnett’s post-test or Student’s *t*-test to compare means, respectively, according to the statistic requirements. Results were considered significant when *p* values were smaller than 0.05.

## 3. Results and Discussion

### 3.1. Screening Rab GTPases for Their Functional and Phenotypic Impacts

Decoding the Rab network that controls the responses of a GPCR may reveal the intermediate steps in its itinerary that are directly linked with its function. Moreover, identification of the Rabs that impact a process such as exocytosis, which relies on granule biogenesis, transport, tethering and fusion, is also likely to reveal the intermediate steps involved in the exocytic process. Therefore, we screened Rab GTPases for their functional impact on MRGPRX2-dependent exocytosis, a process that mediates MC responses during innate immunity, neuroinflammation, and pseudoallergic responses to drugs [35,36]. We chose to perform a gain-of-function screen, which is based on the expression of constitutively active (CA) Rab mutants, in order to avoid the problem of functional redundancy that often masks the involvement of functionally redundant Rabs during a loss-of-function screen, which is based on Rab silencing. In contrast, regardless of potential redundancy in their function, CA Rabs bind their effector proteins, and thereby amplify their regulated step. Indeed, by performing a gain-of-function screening of IgE/Ag-triggered exocytosis, we have previously identified Rabs that control SG fusion, SG transport, and late steps in the exocytic pathway [30]. Since human MCs that express MRGPRX2 are difficult to transfect, we used the RBL-2H3 rat mast cell line as a model that has been stably transfected with the human MRGPRX2 receptor (hereinafter RBL-MRGPRX2 cells) [12]. These cells offer the advantage of higher transfectability as compared to MRGPRX2-expressing human MC models. Moreover, the fact that naïve RBL cells do not express the rodent Mrgprs and therefore lack responsiveness to Mrgpr ligands offers the advantage of exclusive attribution of their acquired responses to MRGPRX2, without the complications that may arise due to the endogenous expression of canonical receptors, such as Neurokinin receptor-1 and -2, which some of these ligands, such as SP, may have [37,38]. Furthermore, we have recently validated the authenticity of this model, by demonstrating that the ectopically expressed MRGPRX2 in RBL cells fully preserves the authentic signaling of the endogenously expressed MRGPRX2 in the human LAD-2 MC line [39]. 

**Figure 1:**
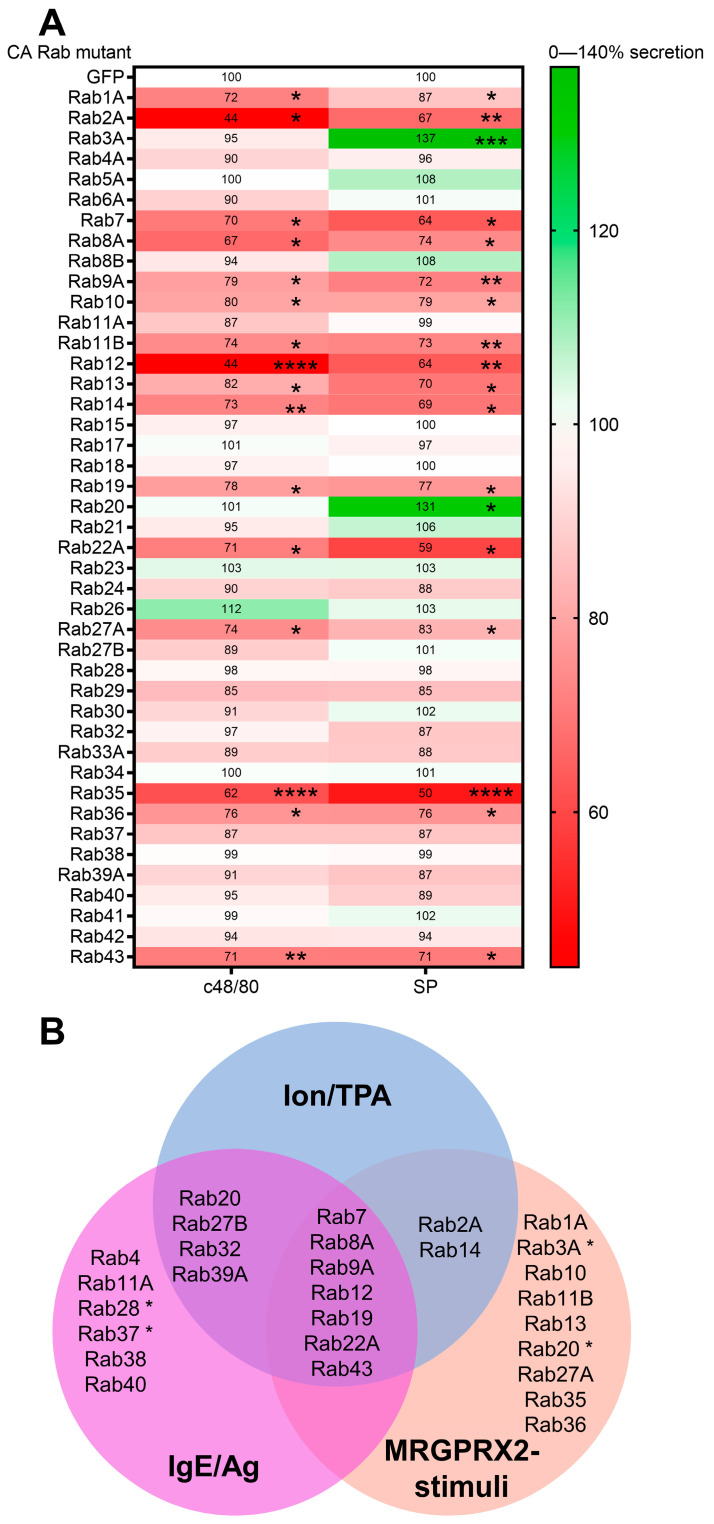
Expression of CA Rab mutants affects MRGPRX2-mediated release of the SG reporter NPY-mRFP. (**A**) RBL-MRGPRX2 cells were co-transfected with 20 μg NPY-mRFP and 30 μg of the indicated pEGFP-CA Rab mutants. Cells were then either left untreated or stimulated for 30 min with either 1 μg/mL c48/80 or 10 μM SP. NPY-mRFP release was measured and compared with release from cells co-expressing NPY-mRFP and pEGFP, set as 100%. Data are the means ± SEM (*n* = 3–8). Statistical significance was determined by one-way ANOVA, followed by Dunnett’s post-test (* *p* < 0.05, ** *p* < 0.01, *** *p* < 0.001, **** *p* < 0.0001). (**B**) Venn diagram of CA Rab mutants that inhibit or potentiate (marked by asterisk) secretion of NPY-mRFP release, which is co-expressed with the Rab mutant, in response to either cell trigger type, or that selectively affect secretion of NPY-mRFP that is induced by either MRGPRX2 stimuli or by Ion/TPA, but not by IgE/Ag, or Rabs that exclusively affect either MRGPRX2 or IgE/Ag-induced secretion of NPY-mRFP.

For screening, we employed our previously described screening method [30], which is based on the co-expression of CA Rab mutants fused to GFP, with Neuropeptide Y fused to monomeric RFP (NPY-mRFP) as a reporter of exocytosis. This method allows the exclusive monitoring of secretion from cells that co-express the Rab mutants, thereby overcoming the background of release from non-transfected cells [30]. We screened forty-three CA mutants of Rabs, which based on our previous results [30], are endogenously expressed in MCs, for their impact on secretion that we triggered with the synthetic polyamine c48/80 or the neuropeptide SP, two established ligands of MRGPRX2. As shown in Figure 1, our screen identified sixteen Rabs, including Rab1A, Rab2A, Rab7, Rab8A, Rab9A, Rab10, Rab11B, Rab12, Rab13, Rab14, Rab19, Rab22A, Rab27A, Rab35, Rab36, and Rab43, which, in their GTP-trapped form, inhibited secretion triggered by either c48/80 or SP, and two Rabs, Rab3A and Rab20, which, in their active conformation, selectively potentiated SP-induced secretion, but not c48/80-induced secretion. Using a gain-of-function approach has the limitation of false positive hits, as CA Rab mutants may acquire non-physiological functions due to their constitutive activation. However, the fact that, out of the forty-three CA Rabs that we screened, twenty-five had no effect on exocytosis suggests that at least in the majority of cases, ectopically expressed CA Rabs do not acquire non-physiological responses. Indeed, our previous gain-of-function Rab screen of IgE-dependent exocytosis identified Rabs, such as Rab5, Rab11, Rab12, Rab27B, and Rab37, whose role in controlling MC exocytosis is established [30,40,41]. Notably, out of the eighteen Rabs that affected MRGPRX2-mediated secretion, seven CA Rabs were found during our previous screen to also inhibit IgE-dependent exocytosis or exocytosis triggered by the combination of a calcium ionophore and 12-O-tetradecanoylphorbol-13-acetate, (Ion/TPA), which stimulates secretion downstream of receptors [42] (Figure 1B). Therefore, this group of ‘general inhibitory Rabs’, comprising Rab7, Rab8A, Rab9, Rab12, Rab19, Rab22A, and Rab43, can be regarded as regulators of the common steps of exocytosis, irrespectively to the type of trigger, while the remaining eleven Rabs are unique to MRGPRX2.

To gain insights into the mechanisms by which positive hits of the screen may influence exocytosis, we leaned on their known functions in other cellular systems and also complemented our functional screening with a phenotypic screen, aimed at identifying morphological changes that might be associated with the Rabs’ functional impact. 

Visualization of the CA-Rab-mutant-expressing cells by confocal microscopy revealed that all of them resided, at least partially, at the SGs under basal or triggered conditions (Supplemental Figure S1). Moreover, with the exception of Rab14 and Rab27A, which had no morphological impact on the cells, the remaining Rabs, which inhibited MRGPRX2-mediated secretion, imposed a specific morphological phenotype, which could be further categorized into an impact on the positioning of the SGs, an impact on the SG size, or an impact on cell morphology. 

**Figure 2:**
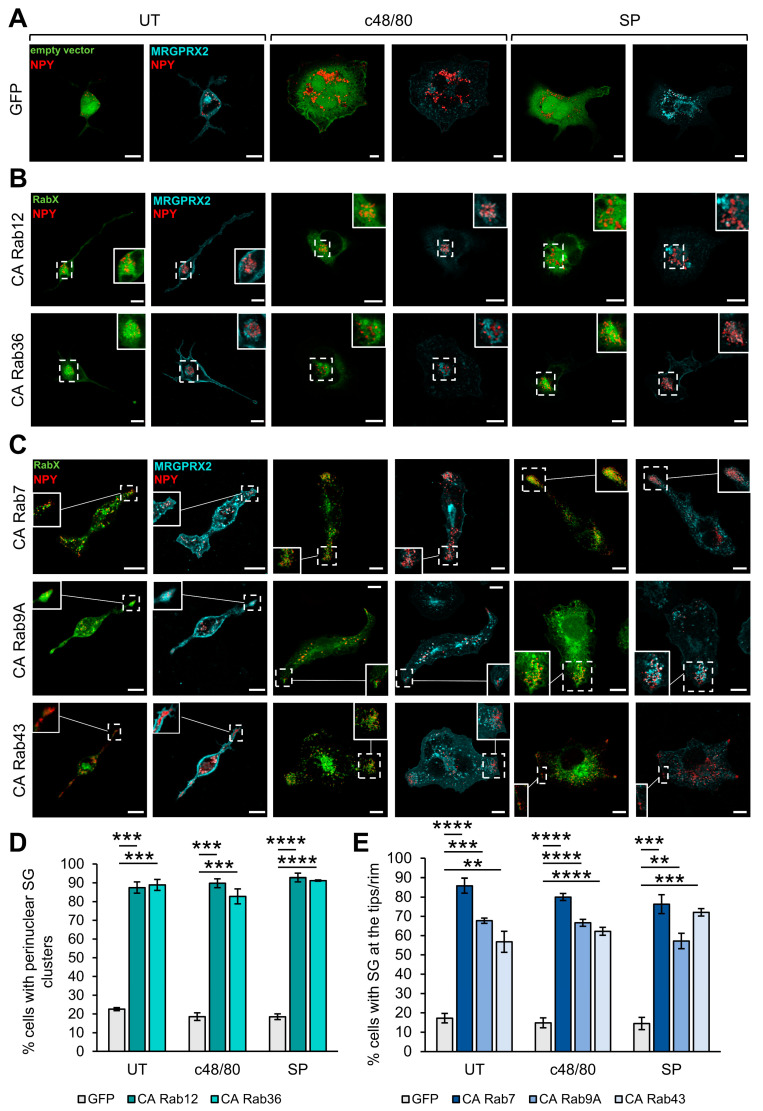
Inhibitory Rabs that affect the SG positioning. RBL-MRGPRX2 cells were co-transfected with 20 μg NPY-mRFP (red) and 30 μg of pEGFP empty vector (**A**), or pEGFP-CA Rab12 or CA Rab36 (**B**), or CA Rab7, CA Rab9A or CA Rab43 (**C**) (green) and either left untreated (UT) or stimulated for 30 min with either 1 μg/mL c48/80 or 10 μM SP, as indicated. Cells were immunostained with anti-HA antibodies followed by Alexa Fluor^®^ 647-conjugated secondary antibodies (pseudo color cyan). Insets are enlargements of the boxed areas. Scale bar = 10 μm. The incidence of cells with perinuclear SGs (**D**) and the incidence of cells with SGs at the cell tips (**E**) were quantified from at least three independent experiments with 15–20 cells in each and are displayed as percentage of total cells. Data are the means ± SEM (*n* = 3–4, 15–20 cells each). Statistical significance was determined by one-way ANOVA, followed by Dunnett’s post-test (** *p* < 0.01, *** *p* < 0.001, **** *p* < 0.0001).

Consistent with our previous findings [30], expression of CA Rab12, which we have previously shown to restrict secretion by recruiting the RILP–dynein complex to the SGs and promoting their retrograde transport [41], led to the perinuclear clustering of the SGs (Figure 2A,B). Quantification of the images revealed that approximately 90% of cells expressing CA Rab12 displayed perinuclear clustering of the SGs (Figure 2D). The SGs also remained clustered in triggered cells (Figure 2B,D) and, accordingly, were prevented from degranulation (Figure 1). Intriguingly, a similar phenotype was noted in cells that expressed CA Rab36 (Figure 2). However, unlike Rab12 which belongs to the group of the ‘general inhibitory Rabs’, CA Rab36 selectively inhibited MRGPRX2-induced secretion (Figure 1B). A possible reason for this discrepancy might be the different modes of exocytosis that are triggered by IgE/antigen (Ag) versus MRGPRX2 [43], and which may also depend on different regulators of SG transport. 

An opposite effect on SG positioning was observed in cells expressing the active mutants of Rab7, Rab9A, and Rab43, in which the SGs accumulated at the cell tips/rim leading to the formation of peripheral SG clusters (Figure 2C,E). Since these clusters localized to the cell tips which were already under basal conditions, we postulate that a “traffic jam” of the SGs, enforced by the expression of these Rab mutants, is the cause rather than the consequence of the inhibition of exocytosis (Figure 1). Indeed, the same Rabs also inhibited secretion induced by either IgE/Ag or Ion/TPA [30] (Figure 1B), while enforcing a similar phenotype. It is interesting to note in this context that local “traffic jams” of synaptic vesicles have been shown to inhibit their release from neurons [44]. We presently do not know how these Rabs promote the accumulation of SGs at cell tips. MC SGs move bidirectionally [45,46]. Therefore, these Rabs may either stimulate SG microtubule-dependent anterograde transport or inhibit their microtubule-dependent retrograde transport. Alternatively, these Rabs may impact the actin cytoskeleton. Rab43 has been implicated in regulating the anterograde transport of GPCRs [47]. However, since this Rab also inhibits secretion induced by IgE/Ag or Ion/TPA, its function in MCs might be unrelated to its impact on GPCR transport.

**Figure 3:**
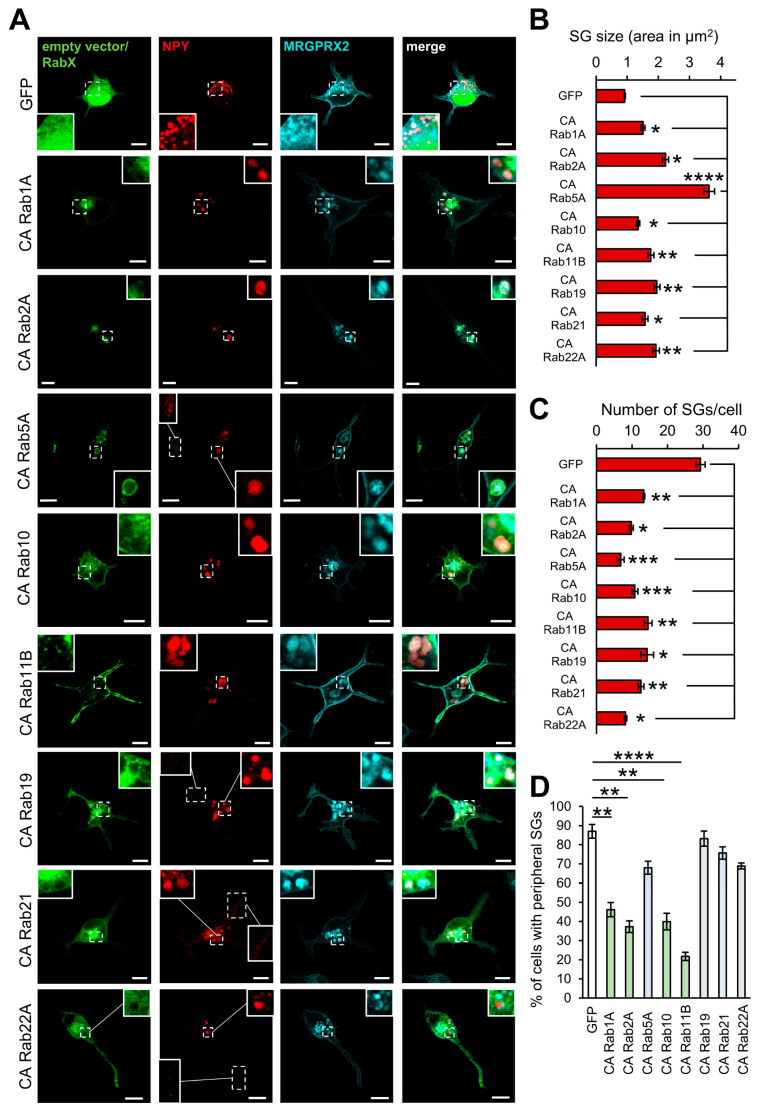
Rabs that enlarge the SGs. RBL-MRGPRX2 cells were co-transfected with 20 μg NPY-mRFP (red) and 30 μg of either empty pEGFP vector or the indicated pEGFP-CA Rab mutants (green). Cells were immunostained with anti-HA antibodies followed by Alexa Fluor^®^ 647-conjugated secondary antibodies (pseudo color cyan) and visualized by confocal fluorescence microscopy (**A**). Insets are enlargements of the boxed areas. Scale bar = 10 μm. The mean SG area (**B**) and the number of SGs/cell (**C**) were quantified using Fiji ImageJ (Version 2.9.0/153t). Cells that contain peripheral SGs were counted and their incidence is presented as percentage of the total cells analyzed in at least three independent experiments with 15–20 cells each (**D**). Data are the means ± SEM (*n* = 3–5, 15–20 cells each). Statistical significance was determined by one-way ANOVA followed by Dunnett’s post-test in (**B**–**D**) (* *p* < 0.05, ** *p* < 0.01, *** *p* < 0.001, **** *p* < 0.0001).

Eight Rabs increased the SG size in the RBL-MRGPRX2 cells. This group included CA Rab5, which we have previously shown to increase the SG size in naïve RBL cells [40]. However, out of these eight Rabs, CA Rab1A, CA Rab2A, CA Rab10, CA Rab11B, CA Rab19, CA Rab21, and CA Rab22A had all significantly and exclusively enlarged the SGs in the MRGPRX2-expressing cells (Figure 3A,B). Given our previous results [25], which documented the presence of MRGPRX2 in the SGs also under basal conditions, the finding that the impact of these Rabs is confined to MRGPRX2-expressing cells strongly implicates MRGPRX2 in the biogenesis of the SGs. The increase in SG size was associated with a significant decrease in the SG number (Figure 3C), suggesting that their increased size was not due to swelling, but involved homotypic SG fusion. With the exception of CA Rab5A and CA Rab21, which had no effect on secretion, all other Rabs also inhibited secretion (Figure 1A). We investigated the possibility that inhibition of secretion was due to the reduced motility of the SGs due to their increase in size. However, quantitative analysis of the confocal images revealed that while cells expressing CA Rab5A or CA Rab21, which did not inhibit secretion, also contained SGs at their cell tips, CA Rab1A, CA Rab2A, CA Rab10, or CA Rab11B, which did inhibit secretion, also had fewer SGs in the cell periphery (Figure 3A,D), suggesting a positive correlation between SG size and SG motility. Exceptions were CA Rab19 or CA Rab22A-expressing cells, which displayed peripheral SGs (Figure 3A,D), despite their inhibition of secretion (Figure 1A). However, these Rabs belong to the family of general inhibitor Rabs, which also inhibit IgE-mediated secretion in naïve RBL cells, although they do not impact the SG size in these cells (Figure 1B). Therefore, their underlying mechanisms of inhibition of secretion is presumably unrelated to their enforced enlargement of the SGs in RBL-MRGPRX2 cells. Interestingly, Rab1A [48,49], Rab2A [50], Rab10 [51,52], Rab11B [53], and Rab19 [54], were all implicated in playing a role in autophagosome formation. Therefore, in view of previous findings that documented the presence of LC3 on MC SGs [55], and our recent findings, according to which SP stimulates SG fusion with autophagosomes [25], we propose that these Rabs increase the SG size by stimulating SG fusion with autophagosomes. 

**Figure 4:**
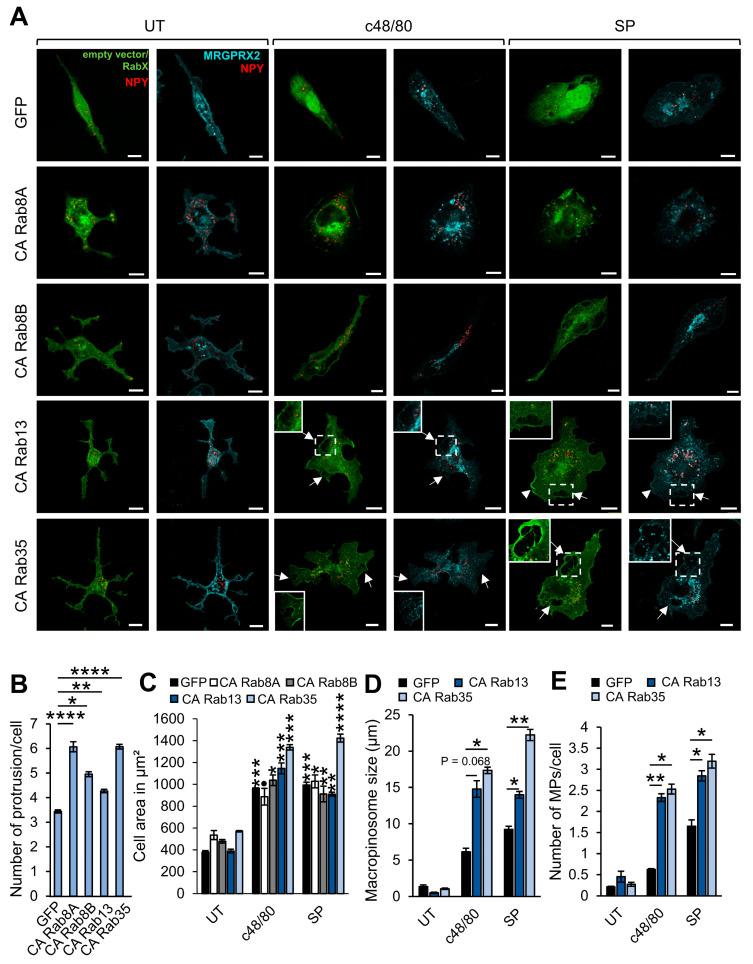
Rabs that affect cell morphology. RBL-MRGPRX2 cells were co-transfected with 20 μg NPY-mRFP (red) and either empty vector or 30 μg of pEGFP-CA Rab8A, CA Rab8B, CA Rab13, or CA Rab35 (green) (**A**). Cells were then either left untreated (UT) or stimulated for 30 min with either 1 μg/mL c48/80 or 10 μM SP, as indicated. Cells were immunostained with anti-HA antibodies followed by Alexa Fluor^®^ 647-conjugated secondary antibodies (pseudo color cyan) and visualized by confocal microscopy. Insets are enlargements of the boxed area. Arrows point to macropinosomes, arrowheads point to macropinocytic cups. Scale bar = 10 μm. The number of protrusions/cell (**B**), the cell area (**C**), the size of macropinosomes (**D**), and number of macropinosomes/cell (**E**) were calculated as described under Materials and Methods. Data are the means ± SEM (*n* = 3–4, with 15–20 cells each). Statistical significance was determined by one-way ANOVA, followed by Dunnett’s post-test (* *p* < 0.05, ** *p* < 0.01, *** *p* < 0.001, **** *p* < 0.001; ● *p* [CA Rab8A: UT vs. c48/80] = 0.1092). The asterisks in (**C**) indicate statistical significance in comparison to their respective untreated control.

In agreement with their role in controlling actin rearrangements [56,57,58,59], CA Rab8A, CA Rab8B, CA Rab13, and in particular CA Rab35 stimulated the formation of numerous protrusions, thereby affecting the cell morphology (Figure 4A,B). However, this change in morphology of the resting cells did not alter their typical flattening in response to activation by either c48/80 or SP (Figure 4A,C). Moreover, although both Rab8A and Rab8B have induced the formation of membrane protrusions, only CA Rab8A affected MRGPRX2-mediated exocytosis (Figure 1A). Therefore, the underlying mechanism of inhibition of secretion by these Rabs may not necessarily be related to the morphological changes that they enforce on resting cells. 

### 3.2. Rabs That Regulate MRGPRX2-Mediated Exocytosis by Controlling Macropinocytosis

**Figure 5:**
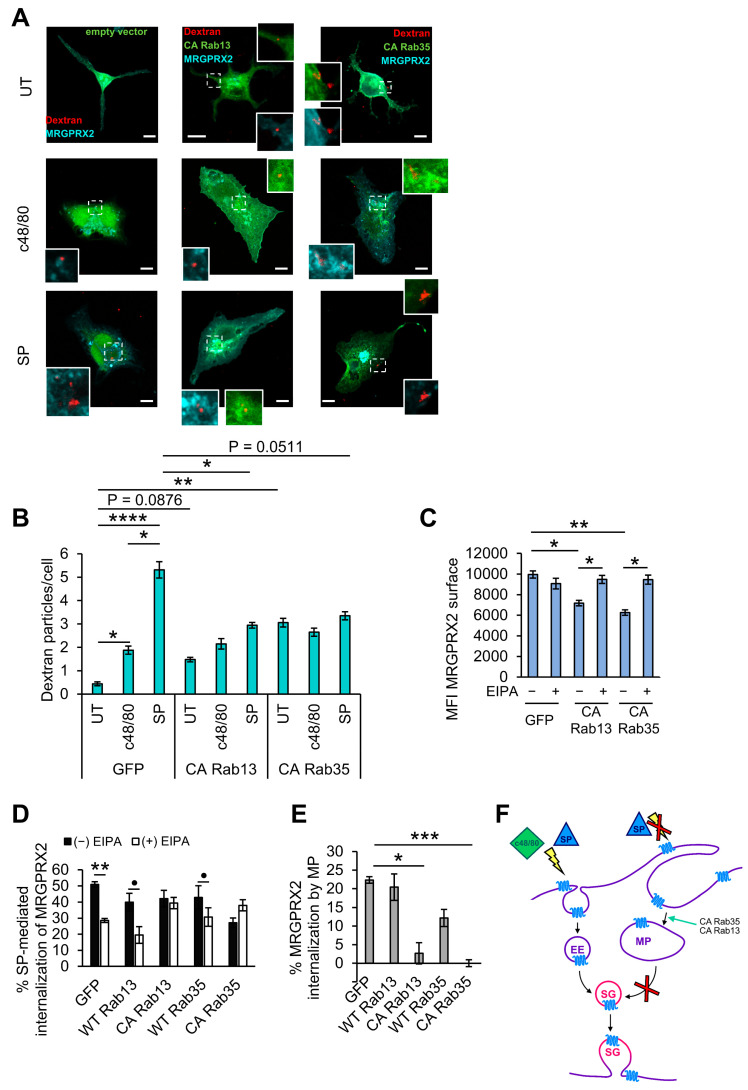
CA Rab13 and CA Rab35 stimulate basal macropinocytosis. RBL-MRGPRX2 cells were transfected with either 30 μg of empty vector or pEGFP-CA Rab13 or pEGFP-CA Rab35 (green). Cells were then incubated for 15 min with 0.1 mg/mL TRITC-Dextran (red) in the absence (UT) or presence of 1 μg/mL c48/80 or 10 μM SP, as indicated (**A**,**B**) or either in absence of trigger or with either 1 μg/mL c48/80 or 10 μM SP in the absence or presence of 50 μM EIPA (**C**–**E**). Cells were immunostained with anti-HA antibodies for confocal microscopy (**A**,**B**) or with anti-MRGPRX2 for flow cytometry to determine the cell surface expression of MRGPRX2 (**C**–**E**) followed by Alexa Fluor^®^ 647-conjugated secondary antibodies (pseudo color cyan). Insets are enlargements of the boxed areas. Scale bar = 10 μm. Dextran uptake was quantified using ImageJ and is presented as the number of Dextran puncta/cell (**B**). The receptor cell surface expression is presented as the Mean Fluorescent Intensity (MFI). The percentage of internalization by macropinocytosis (MP) was defined as the fraction of internalization that is EIPA sensitive (**D**). Negative values are displayed as zero. Data are the means ± SEM [*n* = 2–4, with 10–15 cells each (**A**,**B**), *n* = 4–8 (**C**), *n* = 3–8 (**D**,**E**)]. Statistical significance was determined by one-way ANOVA, followed by Holm–Sidak’s multiple comparison post-test in (**B**,**C**), Student’s *t*-test in (**D**) (** *p* (GFP: (−) EIPA vs. (+) EIPA = 0.0056, ● *p* [WT Rab13: (−) EIPA vs. (+) EIPA] = 0.139, ● *p* [WT Rab13: (−) EIPA vs. (+) EIPA] = 0.16) or Dunnett’s post-test in (**E**) (*p* [UT: GFP vs. CA Rab13] = 0.0876, P[SP: GFP vs. CA Rab35] = 0.0511, * *p* < 0.05, ** *p* < 0.01, *** *p* < 0.001, **** *p* < 0.0001). A schematic presentation of the internalization of MRGPRX2 in response to c48/80 and SP in cells expressing either CA Rab13 or CA Rab35 is presented in (**F**). According to our model, expression of either CA Rab13 or CA Rab35 stimulates basal macropinocytosis. However, because the macropinosomes fail to resolve, marked by the red cross, the internalization of SP-bound MRGPRX2 via macropinocytosis is blocked and shifted to endocytosis.

While in resting cells, the primary phenotype of cells expressing CA Rab13 or CA Rab35 was their increased protrusions, in activated cells, the primary phenotype of these cells was the formation of large macropinosomes, while both Rabs distributed between membrane ruffles, enlarged macropinosomes, and the SGs (Figure 4A). Quantification of the size and number of the macropinosomes revealed a significant increase in macropinosome size and number in cells overexpressing either CA Rab13 or CA Rab35, in cells triggered by either c48/80 or SP (Figure 4D,E). Therefore, in agreement with their reported role [60,61,62], these Rabs seemed to stimulate macropinocytosis, a process which we have recently shown to mediate MRGPRX2 internalization and delivery to the SGs [25]. To substantiate this notion, we directly analyzed the influence of CA Rab13 and CA Rab35 on the uptake of 70 kDa Dextran, a macropinocytosis internalization marker. This analysis indeed demonstrated an increased uptake of dextran in untriggered cells that expressed the active Rab mutants (Figure 5A,B). In the absence of the Rab mutants, both SP and c48/80 stimulated dextran uptake, though SP was significantly more potent than c48/80 (Figure 5A,B). However, expression of either CA Rab13 or CA Rab35 mutants significantly inhibited dextran uptake in SP-activated cells, while the uptake by c48/80-triggered cells was unaffected (Figure 5B). We interpret these results as indicating that hyperactivation of the macropinocytic process by the combined presence of SP and the CA Rab mutants that enhance macropinocytosis exceeds the capacity of macropinosome resolution, which in response elicits an inside-out signal to arrest macropinocytosis. 

Unexpectedly, expression of neither CA Rab13 nor of CA Rab35 led to the entrapment of MRGPRX2 in the enlarged macropinosomes (Figure 4A), though FACS analysis of MRGPRX2 internalization revealed its reduced surface expression (Figure 5C). Furthermore, while in agreement with our previous results [25], treatment of control, GFP-expressing cells with EIPA, an inhibitor of macropinocytosis which inhibits Cdc42 and Rac1-signaling by the targeting of the Na^+^/H^+^ exchanger, NHE1 [63], inhibited SP-stimulated internalization of MRGPRX2 by 50% (Figure 5D); EIPA had no effect on MRGPRX2 internalization in SP-activated cells that expressed either CA Rab13 or CA Rab35 (Figure 5D,E). Therefore, these results imply that inhibition of macropinosome resolution, which is a prerequisite for macropinocytosis progression, shifts the internalization of MRGPRX2 from macropinocytosis to exclusive endocytosis. This shift did not occur in cells expressing WT Rab13 or WT Rab35, in which internalization of SP-bound MRGPRX2 remained EIPA-sensitive (Figure 5D,E), supporting further the connection between inactivation of these Rabs, as a prerequisite for macropinosome resolution, and macropinosome resolution as a prerequisite for propagation of macropinocytosis. Hence, jointly, these results are compatible with a model (Figure 5F), whereby Rab13 and Rab35 actively cycle between their active GTP-bound and inactive GDP-bound conformations, whereby in their active state they stimulate internalization by macropinocytosis, but then need to deactivate and dissociate from the macropinosome to allow its resolution, which allows their reactivation and initiation of another cycle of macropinocytosis. However, in the presence of CA Rab mutants, whose inactivation and dissociation are prevented, macropinosome resolution is arrested, resulting in large macropinosomes alongside the inhibition of macropinocytosis progression. These results thus imply that macropinosome resolution provides a positive feedback control for the succession of the macropinocytic process. These results also predict that macropinosome resolution is the rate-limiting step in the macropinocytic process and implicate that macropinosome resolution is critical for MRGPRX2-stimulated exocytosis. We envision that the enhanced and larger secretory responses that are mediated by MRGPRX2, as compared to IgE-mediated responses, require efficient regranulation by macropinocytosis-mediated recapture of exocytosed SGs and integration of their content into existing SGs, as well as fast recycling of MRGPRX2 from the SGs to the cell surface. 

Surprisingly, unlike SP-stimulated internalization of MRGPRX2, c48/80-triggered internalization of the receptor was also resistant to EIPA in control cells (Supplemental Figure S2A). We have also confirmed in the human LAD-2 MCs, which endogenously express MRGPRX2 (Supplemental Figure S2B), that unlike SP, c48/80 triggers MRGPRX2 internalization predominantly via endocytosis. These results, which are also consistent with the lower potency of c48/80 to stimulate macropinocytosis (Figure 5A,B), add on to our previous results that have already documented differences between MRGPRX2 ligands, categorizing them into ligands, such as SP, that stimulate receptor internalization, and ligands, such as the antimicrobial peptide AG30/5C, that retain the receptor at the plasma membrane [64]. Here, we show that ligands that stimulate receptor internalization can be further categorized into ligands, such as SP, that stimulate receptor internalization by both endocytosis and macropinocytosis, and ligands, such as c48/80, that stimulate receptor internalization by endocytosis. 

### 3.3. Rabs That Selectively Potentiate SP-Induced Secretion

**Figure 6:**
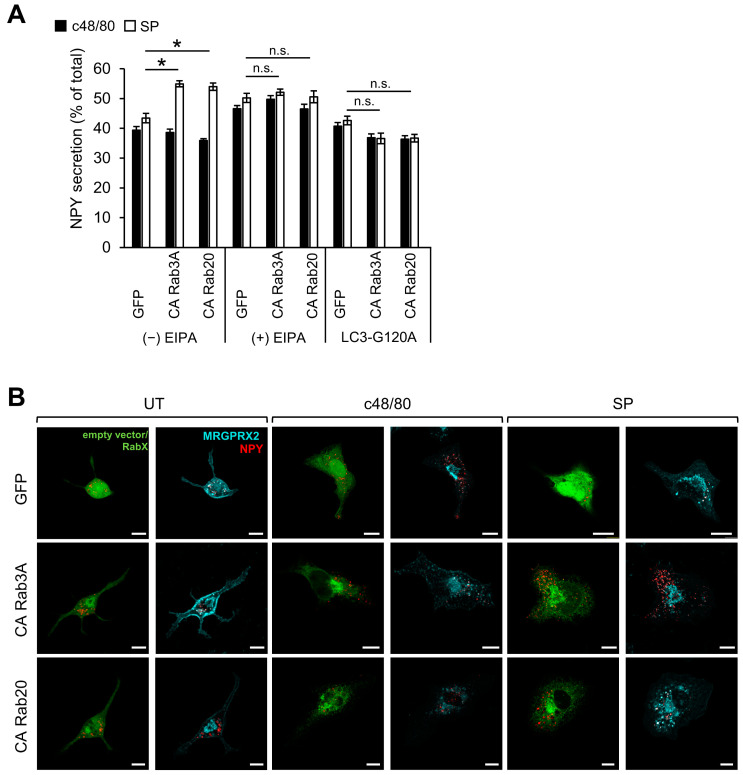
CA Rab3A and CA Rab20 potentiate SP-induced secretion. RBL-MRGPRX2 cells were either co-transfected with 10 μg NPY-mRFP and 15 μg pEGFP-LC3G120A and 15 μg of either empty vector (GFP), or pEGFP-CA Rab3A, or pEGFP-Rab20 (**A**), or co-transfected with 20 μg NPY-mRFP (red) and either 30 μg pEGFP-CA Rab3A or pEGFP-CA Rab20 (green) (**B**). Cells were then either left untreated or pre-incubated for 30 min with 50 μM EIPA, and then stimulated for 30 min with either 1 μg/mL c48/80 or 10 μM SP as indicated (**A**,**B**). Release of NPY-mRFP is presented as percentage of total (**A**). Cells were immunostained with a monoclonal anti-HA antibody followed by Goat Anti-Mouse IgG H&L (Alexa Fluor^®^ 647) (pseudo color cyan) (**B**). Insets are enlargements of the boxed areas. Scale bar = 10 μm. Data are the means ± SEM [*n* = 6–7 in (**A**) and *n* = 3–4, 15–20 cells each in (**B**)]. Statistical significance was determined by one-way ANOVA, followed by Dunnett’s post-test (* *p* < 0.05, n.s. (= not significant) ≥ 0.05).

While the majority of Rabs had a similar impact on either c48/80 or SP-stimulated secretion, expression of either CA Rab3A or CA Rab20 selectively potentiated MRGPRX2-mediated release that was triggered by SP (Figure 1A). Since only SP, but not c48/80, stimulates MRGPRX2 internalization via macropinocytosis, we suspected that Rab3A and Rab20 may affect a step in the macropinocytic trafficking of the receptor. Therefore, we asked if EIPA would impact the ability of these Rabs to potentiate SP-induced secretion. Indeed, in the presence of EIPA, neither CA Rab3A nor CA Rab20 recapitulated their stimulatory impact on SP-induced secretion (Figure 6A). Since the expression of these Rabs was not linked with an apparent increase in macropinosome number or size (Figure 6B), we reasoned that they may regulate the resolution of macropinosomes, which, according to our model (Figure 5C), is the rate limiting step in macropinocytosis. Since we have previously shown that this step involves the recruitment of LC3 [25], we investigated how the expression LC3-G120A, a lipidation-deficient mutant of LC3 [65], would influence secretion. By itself, overexpression of LC3-G120A did not influence degranulation (Figure 6A). However, the expression of this mutant eliminated the ability of either CA Rab3A or CA Rab20 to enhance SP-induced secretion (Figure 6A). These results are consistent with the positive regulation of macropinocytosis by macropinosome resolution. Hence, inhibition of the latter by the expression of the LC3 mutant shifts exocytosis to the macropinocytosis-independent pathway, same as in the presence of CA Rab13 or CA Rab35 (see Model Figure 5C). Therefore, while secretion is not affected, neither CA Rab3A nor CA Rab20 potentiate secretion.

**Figure 7:**
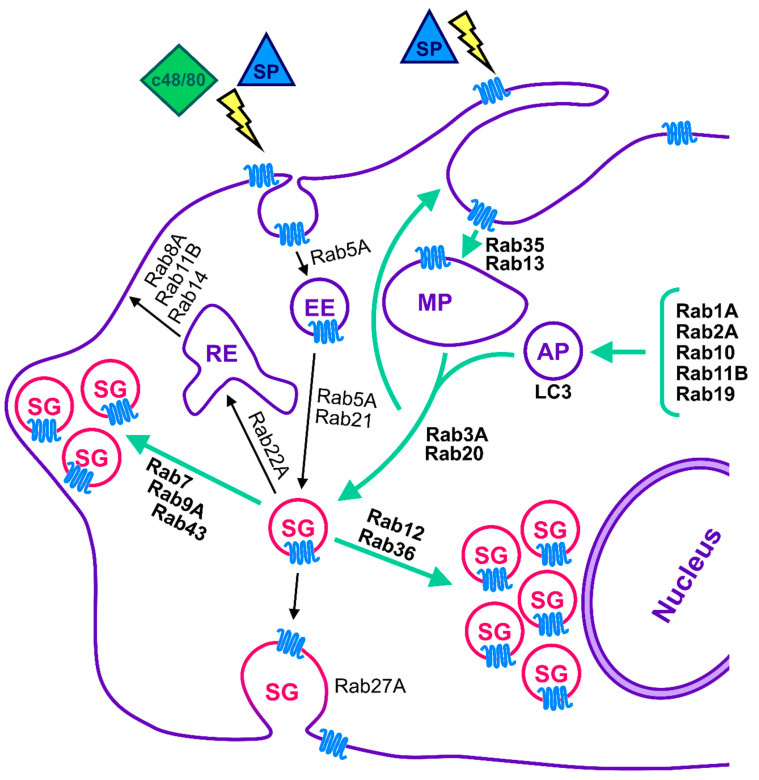
Model for the regulation of MRGPRX2-mediated secretion by Rab GTPases. According to our model, MRGPRX2-mediated secretion is regulated by Rab GTPases that also control both IgE/Ag and Ion/TPA-dependent secretion, but also by unique Rabs. The latter may reflect the distinct modes of exocytosis that are triggered by the distinct stimuli [43] or the influence of MRGPRX2 route of trafficking, which we have previously shown to include, in addition to endocytosis, macropinocytosis and fusion with autophagosomes [25] on its triggered responses. Green arrows indicate the Rab-regulated pathways that stand out as modulators of MRGPRX2-mediated exocytosis. These pathways include the bidirectional transport of the SGs, their plus end transport is influenced by Rab7, Rab9A, and Rab 43, whose constitutive activation results in SG accumulation at cell tips, and their minus end transport that is regulated by Rab12 and Rab36, whose constitutive activation leads to perinuclear accumulation of the SGs. The second pathway, regulated by Rab1A, Rab2A, Rab3A, Rab10, Rab11B, Rab13, Rab19, Rab20, and Rab35 reveals the novel feedback association between macropinocytosis and macropinosome resolution, the close association between macropinocytosis and exocytosis, and the role of autophagosome formation in macropinosome resolution and SG enlargement. Also indicated are Rabs that were screened in this study, and whose functions are based on literature reports.

In summary, our results reinforce the idea that distinct mechanisms couple allergic, i.e., IgE-mediated, and innate, i.e., MRGPRX2-mediated, stimuli with secretion. Our results also imply that while MRGPRX2 binds multiple ligands, they may differ in their underlying mechanisms of action and therefore also in their elicited biological responses, a notion that should be considered in future studies. Focusing on MRGPRX2, our data identified two cellular pathways, whose regulation is critical for MRGPRX2-mediated exocytosis (see Model Figure 7). First is the bidirectional movement of the SGs, where perturbation of either the minus end or plus end transport led to the inhibition of exocytosis. We also identified Rab12 and Rab36 as regulators of the minus end transport of the SGs, and Rab7, Rab9A, and Rab43 as regulators of their plus end movement. The second pathway that emerges as critical for MRGPRX2-mediated exocytosis is macropinosome resolution. Perturbation of this process, either by amplification of macropinocytosis or by inhibition of macropinosome resolution, inhibit exocytosis. The former is caused by the constitutive activation of Rab13 and Rab35, whereas the latter, which is regulated by Rab3A and Rab20, is caused by the inhibition of LC3 lipidation (see model Figure 7). We propose that SG regranulation and/or the coupling of MRGPRX2 recycling to exocytosis, by its delivery to the SG, are crucial for MRGPRX2-mediated secretion. Finally, in agreement with the role of LC3 in macropinosome resolution and SG regranulation, we show that Rabs that stimulate autophagy increase the SG size (Figure 7). Questions that remain open include the mechanism by which Rab7, Rab9A, and Rab43 promote SG accumulation at cell tips, the mechanisms by which Rab22A and Rab14 affect exocytosis, the former regardless of the trigger type and the latter in exocytosis that is selectively triggered by MRGPRX2 ligands, and finally the reason for the isoform type specificity of Rab27, whereby Rab27B controls IgE- and Ion/TPA-stimulated secretion, whereas Rab27A controls MRGPRX2-mediated release. Bidirectional movement of the SGs may be regulated by a “tug-of-war’’ mechanism between oppositely directed molecular motors. Thus, it will be interesting to compare the patterns of SG association with dynein and kinesin-1 (KIF5B), which have been shown to regulate SG transport [66] of control cells and cells that express CA Rab mutants that impact SG positioning. Hence, unlike Rab12 and Rab36 which, by the binding of the RILP–dynein complex, drive the SGs to the minus end, both Rab7 and Rab9 have been implicated in controlling minus end transport of endocytic organelles [67,68], whereas Rab43 was shown to redistribute the p150^Glued^ subunit of dynactin, which is part of the dynein complex [69]. Therefore, their hyperactivation might lead to dynein sequestration, which, by impairing the balance of the bidirectional motility of the SGs, would result in their peripheral accumulation. Both Rab22A and Rab14 play a role in endocytic recycling [70,71] and may impact the recycling of the exocytic machinery. Finally, the Rab27 isoform preference is intriguing. However, MCs release their SG content by different modes of exocytosis, which also bear distinct physiological consequences [43]. MRGPRX2-stimulated release involves the rapid secretion of individual SGs, whereas activation of MCs by IgE/Ag results in the slower exteriorization of groups of SGs formed by the prior fusion of individual SGs, resulting in compound exocytosis. Therefore, the differential regulation of exocytosis by Rab27 isoforms might be related to the distinct modes of exocytosis. Given the ability of GPCRs to elicit distinct signaling when localized to the plasma membrane, or intracellular sites [22,23,24], dissecting the itinerary of a GPCR is critical for decoding its spatiotemporally discrete signaling processes. In particular, a GPCR, such as MRGPRX2, that binds multiple ligands, may elicit distinct ligand-dependent responses, as a function of its ligand-enforced itinerary. Therefore, identifying the steps in MRGPRX2 traffic and the proteins that control its itinerary open opportunities for the development of drugs that would selectively target site and therefore ligand specific undesired responses.

## Data Availability

Data is contained within this article and Supplementary Material.

## Supplementary Materials

The following supporting information can be downloaded at https://www.mdpi.com/article/10.3390/cells13010093/s1: Figure S1: The cellular location of CA Rab mutants that impact MRGPRX2-mediated exocytosis; Figure S2: c48/80-mediated internalization of MRGPRX2 is EIPA-insensitive.
