# Supplementary material for: The Underlying Rab Network of MRGPRX2-Stimulated Secretion Unveils the Impact of Receptor Trafficking on Secretory Granule Biogenesis and Secretion
Source: Cells. 2024 Jan 1;13(1):93. doi: 10.3390/cells13010093 (PMC10778293; doi:10.3390/cells13010093)
Supplement: Supplementary file 1 [file cells-13-00093-s001.zip › cells-2779407-supplementary.pdf]

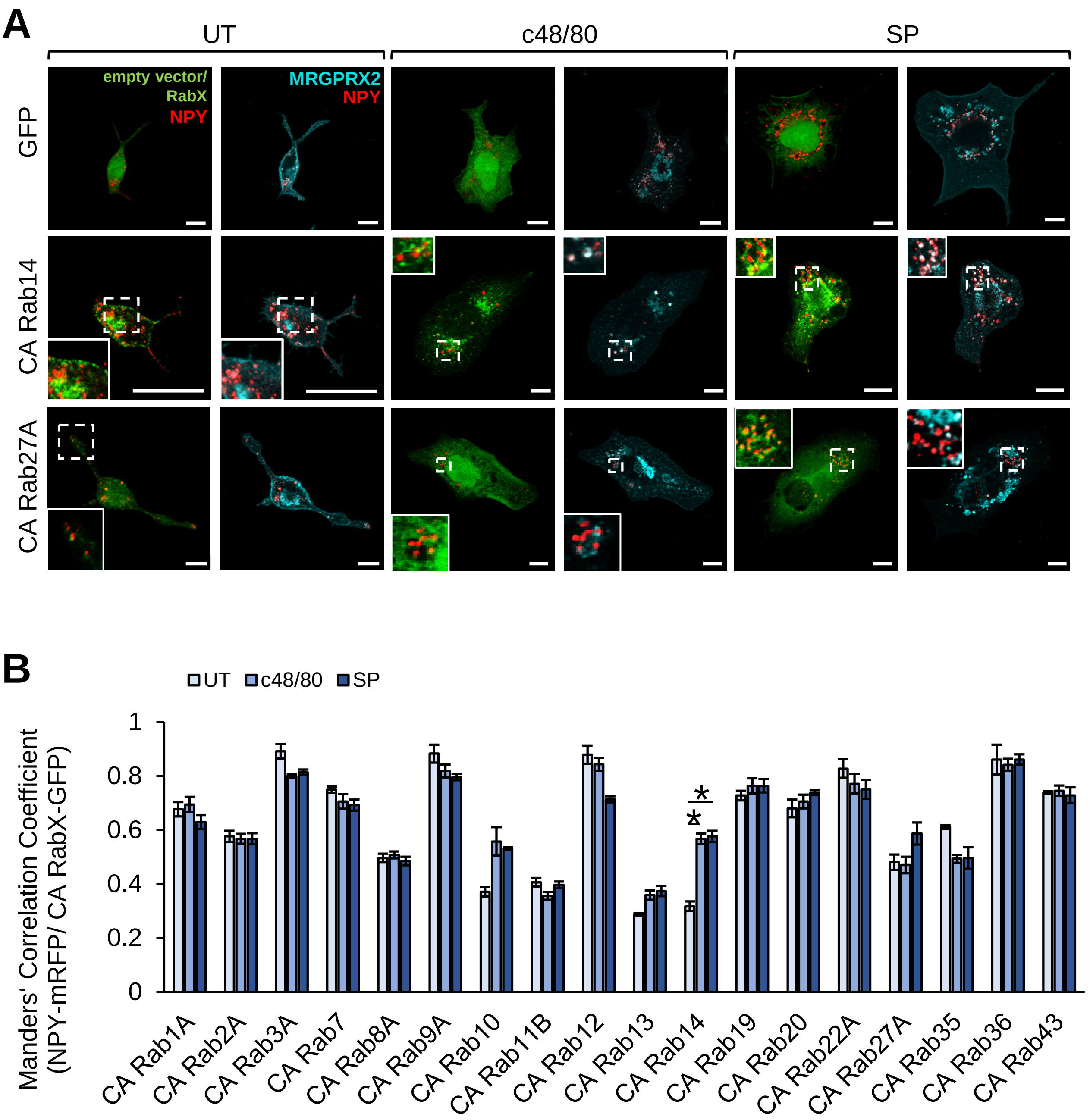

Suppl. Figure S1

Suppl. Figure S1 The cellular location of CA Rab mutants that impact MRGPRX2-mediated exocytosis. RBL-MRGPRX2 cells were co-transfected with 20 µg NPY-mRFP (red) and 30 µg of either empty pEGFP vector (green) or the indicated pEGFP-CA Rab mutants (A, B). Cells were then either left untreated (UT) or stimulated for 30 min with either 1 µg/ml c48/80 or 10 µM SP, and immunostained with anti-HA antibodies followed by Alexa Fluor® 647-conjugated secondary antibodies (cyan) and visualized by confocal microscopy. The extent of overlap of NPY-mRFP with the indicated CA Rab mutant was quantified using the JACoP plugin of the extended ImageJ version Fiji and is presented as Manders' colocalization coefficient (B). Data are the means ± SEM (n = 3-4, 15-20 cells each). The cellular location of CA Rab14 and CA Rab27A is shown in (A). Scale bar = 10 µm. The cellular location of Rabs that affected the cell morphology is shown in the following figures. * $p < 0.05$.

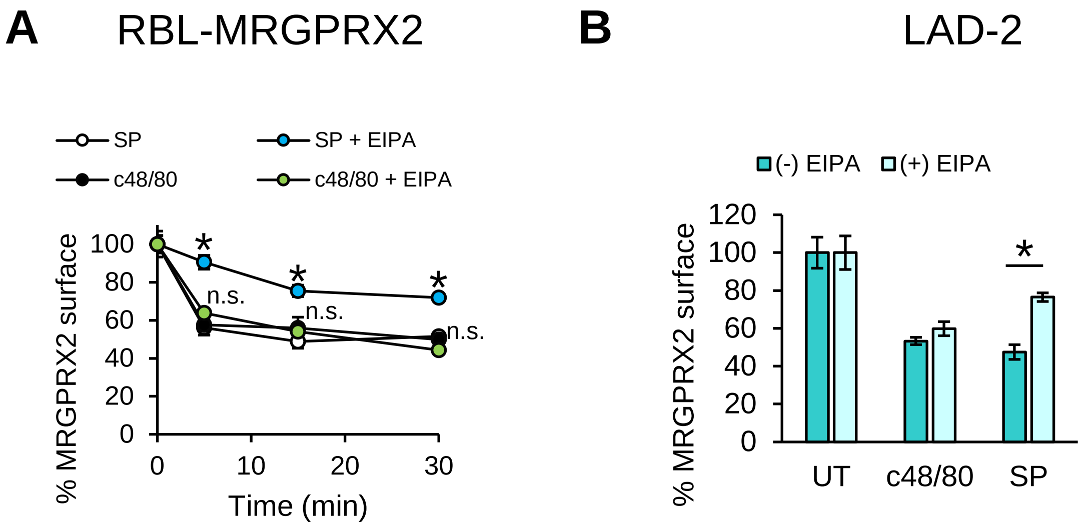

Suppl. Figure S2 c48/80-mediated internalization of MRGPRX2 is EIPA-insensitive. RBL-MRGPRX2 cells (A) or LAD-2 cells (B) were triggered with either 1 µg/ml c48/80 or 10 µM SP in the absence or presence of 50 µM EIPA for 0, 5, 15 min (A) or 30 min (A, B). Cell surface expression was determined by flow cytometry analysis after staining with anti-MRGPRX2 antibodies followed by Alexa Fluor® 647-conjugated secondary antibodies and is presented as the percentage of cell surface MRGPRX2 in the absence of trigger (time 0, UT). Data are the means ± SEM (n = 4). Statistical significance was determined by unpaired two-tailed Student's t-test (A: **P*[5min: (-)SP vs. (+)SP] = 0.0242, **P*[15min: (-)SP vs. (+)SP] = 0.038, **P*[30min: (-)SP vs. (+)SP] = 0.0147; B: *P[SP: (-)EIPA vs. (+)EIPA] = 0.014768.
